# Analysis of the northern pitcher plant (*Sarracenia purpurea* L.) phytotelm bacteriome throughout a temperate region growing season

**DOI:** 10.1371/journal.pone.0306602

**Published:** 2024-07-12

**Authors:** Paul P. Melchior, Emma Reiss, Zachary Payne, Nhi Vuong, Kari Hovorka, Hunter L. Lindsay, Gerardo R. Diaz, Tara Gaire, Noelle Noyes

**Affiliations:** 1 Department of Biology, North Hennepin Community College, Brooklyn Park, Minnesota, United States of America; 2 Department of Biology, Bemidji State University, Bemidji, Minnesota, United States of America; 3 Department of Marine Science, Atlantic Technological University, Galway, Republic of Ireland; 4 Department of Veterinary Medicine, University of Minnesota, St. Paul, Minnesota, United States of America; Universidade de Coimbra, PORTUGAL

## Abstract

The insectivorous Northern Pitcher Plant, *Sarracenia purpurea*, recruits a dynamic biotic community in the rainwater collected by its pitcher-shaped leaves. Insect capture and degradation within the pitcher fluid (phytotelma) has been well documented as a mechanism for supplementing the plant’s nitrogen, phosphorous, and micronutrient requirements. Metagenomic studies have shown a diverse microbiome in this phytotelm environment, including taxa that contribute metabolically to prey digestion. In this investigation, we used high-throughput 16S rDNA sequencing and bioinformatics to analyze the *S*. *purpurea* phytotelm bacteriome as it changes through the growing season (May–September) in plants from the north-central region of the species’ native range. Additionally, we used molecular techniques to detect and quantify bacterial nitrogenase genes (nifH) in all phytotelm samples to explore the hypothesis that diazotrophy is an additional mechanism of supplying biologically available nitrogen to *S*. *purpurea*. The results of this study indicate that while prokaryote diversity remains relatively stable in plants at different locations within our region, diversity changes significantly as the growing season progresses. Furthermore, nifH genes were detected at biologically significant concentrations in one hundred percent of samples, suggesting that nitrogen fixation may be an important contributor to the *S*. *purpurea* nutrient budget.

## Introduction

The northern pitcher plant, *Sarracenia purpurea* ssp. *purpurea* L., is an insectivorous hydrophyte native to nutrient-poor peatlands of North America. The southern fringe of its range in central North America currently extends south from Ontario into central Minnesota, Wisconsin, and Michigan, USA [1,2]. Upon maturation and opening, *S*. *purpurea* leaves, traditionally and hereafter referred to as pitchers because of their shape and function, collect and hold water (i.e. the leaf phytotelma), and capture and degrade invertebrate prey species [3–6]. Pitchers in temperate regions survive overwintering, and are capable of functioning for multiple years [7].

Phytotelmata rapidly recruit inquiline communities of prokaryotes and eukaryotes, generating a unique microecosystem [8–10]. The phytotelm microbiome of *S*. *purpurea* and its congeners has been the subject of numerous studies that describe its composition and diversity. Early culture-based analyses [9,11–14] provided an initial picture of the *S*. *purpurea* phytotelm bacterial community. However, cloning-based 16S rDNA sequence studies quickly revealed a much more extensive microbiome and provided early evidence for its ecological role within the host plant [15,16]. Recent high-throughput 16S rDNA metagenomic studies have allowed a much deeper and broader understanding of these inquiline communities in *S*. *purpurea* and its congeners [17–19]. More broadly, phytotelm metagenome studies in two other families of pitcher-bearing plants, the Nepenthaceae and Cephalotaceae, recently provided compelling evidence for ecological and evolutionary convergence as primary drivers of the composition and function of these microbial communities [20–23].

The degradation and use of arthropod prey by *S*. *purpurea* is accomplished by a variety of mechanisms, including a complex pool of digestive enzymes in the phytotelm fluid. The host plant’s contribution to this enzyme assemblage remains unclear, but appears to be limited [24–26]. Chitinases, acid phosphatases, proteases and other enzyme classes generated by the phytotelm microbiome appear to comprise the bulk of the digestive repertoire, and significantly improve prey decomposition and nutrient availability for the host plants [7,8,27–29]. Dissolved nitrates and ammonia also make their way into pitchers through precipitation deposition [30]. However, researchers have suggested that such exogenous sources of biologically available nitrogen may not completely satisfy the requirements of *S*. *purpurea* [31,32].

Acetylene reduction assays, coupled with the isolation of *Azotobacter* and *Azomonas* species from the *S*. *purpurea* phytotelm fluid, provided early evidence that nitrogen-fixation occurs in these plants [33,34]. Diazotrophic taxa have also been identified as inhabitants of the rhizosphere of *Sarracenia* species [35]. The widespread presence of bacterial nitrogenase genes in the phytotelmata of *Nepenthes mirabilis* (Family Nepenthaceae) adds support to the hypothesis that diazotrophic bacteria play a role in host plant nitrogen acquisition across multiple pitcher plant taxa [36].

In this study we used high-throughput 16S rDNA sequencing to characterize the phytotelm bacteriome of *S*. *purpurea* over the growing season in plants from three isolated bogs at the southern edge of the species’ range in central North America, with a focus on the abundance and prevalence of diazotrophs. In addition, we used qPCR to detect and quantify the bacterial nitrogenase (reductase) gene, nifH in the phytotelm fluid to better define the presence of diazotrophs in this system.

## Materials and methods

### Site selection

**Fig 1:**
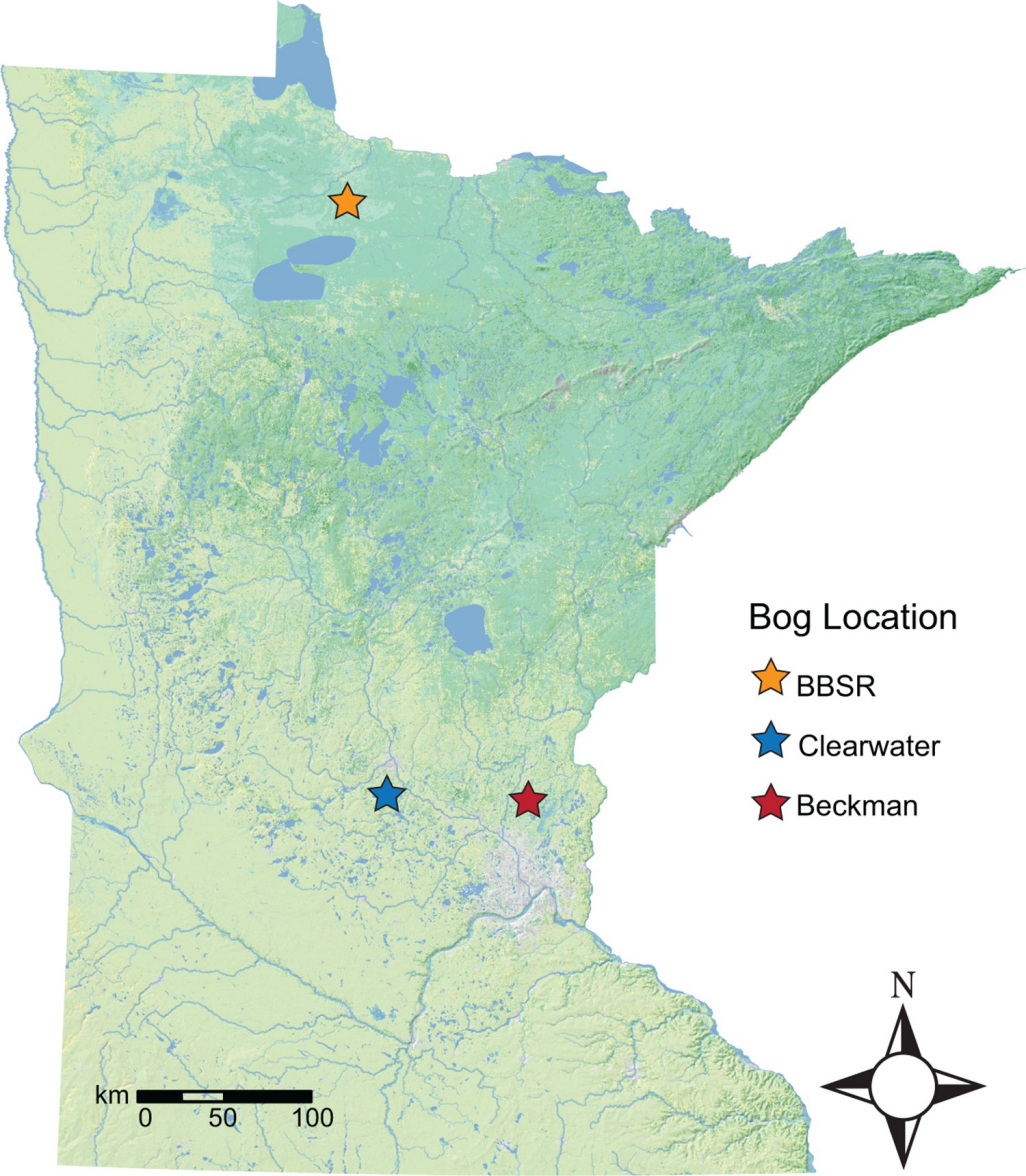
Sample (bog) locations in Minnesota, USA.

Three acidic peatlands at the southern edge of *S*. *purpurea*’s range in Minnesota, USA (Fig 1) were selected as sample sites (hereafter, bog locations), including Big Bog State Recreation Area (BBSR) near the Minnesota-Canada border in Beltrami County, Minnesota (48° 17’ 59.7" N, 94° 34’ 05.1" W), a private bog (Clearwater) in Stearns County, Minnesota (45° 23’ 22” N, 94° 08’ 35” W), and Beckman Lake bog (Beckman), Isanti County in east central Minnesota (45°25’ 18.18” N, 93° 11’12.67” W). BBSR and Clearwater bogs were selected because of their relatively pristine condition and isolation from anthropogenic land use. Beckman bog was chosen because it represents one of the southern-most populations of *S*. *purpurea* in the region, and is closer to anthropogenic disturbances of the landscape. Since removal of plant tissues was not required for this project, no collection permits were required by the Minnesota Department of Natural Resources for the two public land/water sites (BBSR and Beckman), nor for Clearwater bog on private property. BBSR and Beckman sites are open to the public and require no access permits. Land owner permission for access to the Clearwater site was acquired prior to entry.

### Sample collection and preservation

Two plants, approximately 150 m apart, were selected and marked (labelled tag on adjacent shrub) in each bog. These plants were selected because they were representative of the local population, had multiple functional pitchers from the current and previous growing seasons, and were located at least 50 meters from human trails or boardwalks. GPS coordinates for each plant were recorded for subsequent sampling. Three or four functional pitchers from each plant were selected and aseptically marked on the anterior leaf rib. Only intact pitchers with full phytotelmata and no visible dead or damaged leaf tissue were selected. In total, 24 pitchers were each sampled four times (BBSR n = 8; Clearwater n = 10; Beckman n = 6). Two additional pitchers from Beckman Bog (plant B) were initially sampled in May. Subsequent damage to these pitchers prevented further sampling, and the May samples from them were eliminated from this study. In addition, two samples from the BBSR bog (leafID BBSR_B_4 in July, and leafID BBSR_A_1 in September) were damaged during transport, which reduced the total number of samples to 94. The age of each pitcher (pitcher year) was recorded as either newly emerged (first year) or as a functional, previous season leaf (> first year).

The phytotelm fluid from each of the pitchers was mixed with a sterile Pasteur pipette. A three mL sample was removed and transferred to a sterile collection tube and immediately flash-frozen in a -78°C dry-ice/propanol bath. Frozen samples were transported to the lab for storage at -80°C until processing. This method was repeated on all marked pitchers on each of the four sample dates (May 25/June 3 (“May”), July 14 (“July”), August 20 (“August”), and September 15 (“September”), totaling 94 samples for the study.

### DNA extraction and purification

DNA from phytotelm samples and qPCR controls was extracted using QiaAMP BiOstic DNA (product 12240–50) extraction kit and protocol (Qiagen Sciences Inc., Germantown, MD). After consultation with Qiagen staff, this product was chosen because of its excellent direct recovery of bacterial cells from low population density samples. Purified DNA concentration was confirmed by UV spectrophotometry (Thermo Scientific NanoDrop 2000).

### qPCR detection and quantification of nifH genes

Detection and quantification of nifH gene was performed on a PikoReal® qPCR System (Thermo Scientific) using the degenerate IGK3/DVV primer set [37] manufactured by IDT Inc., Coralville, IA. nifH gene copy concentration in phytotelm samples was determined using the standard curve method. A three-replicate nifH qPCR standard curve was generated from a log-growth phase (96 hour) culture of *Rhodospirillum rubrum ATCC 11170* grown in tryptic soy broth at 30°C in a shaker-incubator. Serial 10-fold dilutions of the culture were made, and 100 μl aliquots of each were spread on tryptic soy agar plates. Colonies were counted after incubating plates for 96 h at 30°C. The dilution series and viable plate-count procedure were done in parallel and in triplicate.

Each 20 μL qPCR reaction included 1X GoTaq® SYBR Green qPCR Master Mix (Promega, Madison, WI), 1 μL DNA template, nuclease-free water, 1.0 μM MgCl_2_, and forward and reverse primers at final reaction concentrations of 0.8 μM each. Thermocycling included a 2 min initial denaturation step at 95°C followed by 40 cycles (95°C denature 30 sec; 58°C anneal 30 sec; 72°C elongate 30 sec).

Each qPCR plate included replicates of field sample template DNA, as well as non-template (NTC) controls, negative bacterial DNA controls (purified *Escherichia coli* K12 gDNA), and one-point calibration (OPC) standard/calibrator (*R*. *rubrum* DNA log_10_ = 3.57 nifH copies/reaction). Data was recorded with PikoReal™ software version 2.1 (Thermo Scientific) and exported to Microsoft Excel and R for analysis. Normalization of nifH quantities was done using the OPC method, with standards determined by the standard curve method [38,39]

### 16S DNA sequencing

Sequencing of 16S variable region 4 (V4) for each sample was performed by the University of Minnesota Genomic Center (UMGC), St. Paul, Minnesota. Linker-primers 5’-GTGCCAGCMGCCGCGGTAA and 5’- GGACTACHVGGGTWTCTAAT were used for 2 X 300 bp sequencing on an Illumina MiSeq® sequencer using proprietary adapters and UMGC protocols [40].

### 16S sequence processing and bioinformatics

V4-region primers were removed from sequences with *cutadapt* version 4 [41] using the package default settings. Trimmed 16S fastq files were processed in R version 4.3.0 [42] using *DADA2* version 1.28.0 [43]. DADA2 was used for quality filtering of sequence reads, with the following settings: truncLen = c(240, 160), maxN = 0, maxEE = c(2, 2), truncQ = 2. Quality-controlled, paired-end reads were joined and chimeras removed before amplicon sequence variants (ASVs) were determined. Taxonomic classification of ASVs was performed using *DADA2*’s naïve-Bayesian classifier algorithm incorporating the Silva rDNA reference database version 138.1 [44]. A count matrix of ASVs, sample IDs and read counts were produced and merged with metadata and taxa tables using *phyloseq* version 1.44.0 [45], from which contaminant sequences were identified and removed using the frequency-based method of *decontam* version 1.20.0 for R [46].

### Statistical analysis

The decontaminated count matrix (*phyloseq* object) was used to calculate richness, Shannon diversity, and Pielou’s evenness indices for ASV and higher taxonomic levels. Omnibus mixed effects linear models were generated using *lme4* version 1.1.33 [47] in R to analyze the influence of multiple fixed-effect variables (sample month, bog location, 16S amplicon copies, and pitcher year) and a single random-effect variable (pitcher identity) on taxon richness, Shannon diversity, and Pielou’s evenness indices, as well as nifH gene copy concentration. An interaction term between sample month and bog location was included to allow for potentially differing effects by location and month. The nesting of pitchers within plants was considered as a nested random effects structure, however this model specification produced a singularity warning, and therefore a simpler random effects structure was used to avoid overfitting. Pitcher identity was chosen over plant identity given the self-contained nature of each pitcher, and the fact that sample-level observations were obtained from individual pitchers. Model outputs were analyzed by Type III ANOVA, and estimated marginal means generated for post-hoc, pair-wise Tukey comparison testing were calculated with *emmeans* version 1.8.6 [48] for R.

Non-metric dimensional scaling (NMDS) ordination plots of Bray-Curtis dissimilarity indices calculated from non-normalized ASV counts were generated by *phyloseq*, and samples were visualized by both sample date and bog location. To estimate the proportion of variance in Bray-Curtis dissimilarity values apportioned to bog location and date, we used PERMANOVA testing as implemented in *vegan* version 2.6–4 [49]. Beta dispersion around centroids was calculated with *vegan’s* betadisper function.

Differential abundance testing was performed at the family level with *MaAsLin2* version 1.14.1 [50] on count data normalized by cumulative sum scaling (CSS) with *metagenomeSeq* version 1.42.0 [51]. Model parameters included bog location and sample date as fixed effects, and pitcher identity as a random effect, with minimum prevalence and abundance thresholds set at 0.1 and 0.01, respectively. Relative abundances were compared between bog locations, as well as by sample date. For the latter, July, August, and September were each compared to May to contrast the phytotelm bacteriome as the growing season progressed. All plots were produced with *ggplot2* version 3.4.2 [52] and core R functions.

## Results

### DNA sequencing and analysis

16S-V4 rDNA sequencing of *S*. *purpurea* phytotelm samples (n = 94) generated 3.3 x 10^6^ total paired-end (PE) reads (mean = 4.4 x 10^4^ per sample; range = 1.6 x 10^4^ to 6.6 x 10^4^). After trimming, filtering and chimera removal, 3.17 x 10^6^ PE reads (95.9%) were classified at the ASV level, resulting in 5,245 unique ASVs representing 495 different genera across 233 families. The proportion of pair-end reads classified at each taxonomic rank is illustrated in Table 1.

**Table 1:** Percentage of pair-end reads classified at each taxon.

Taxon	No. PE Reads	Percent Classified
Kingdom	3,168,021	99.93%
Phylum	3,152,872	99.46%
Class	3,150,720	99.39%
Order	3,116,484	98.31%
Family	2,941,778	92.80%
Genus	2,469,052	77.89%
Species	239,417	7.55%

### Alpha diversity

**Fig 2:**
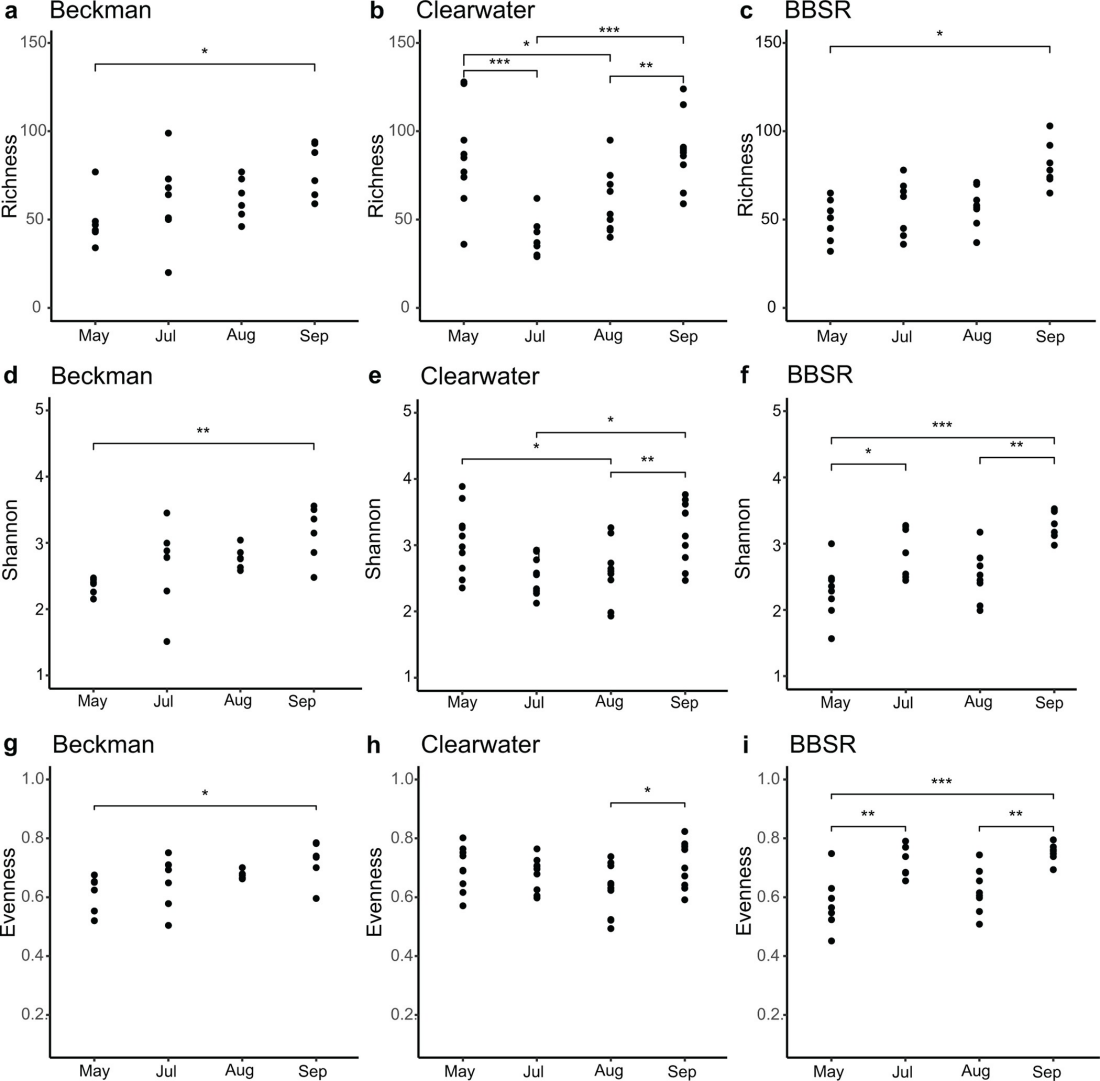
Phytotelm bacteriome alpha diversity (family-level) indices. By bog location [(a—c) richness, (d—f) Shannon diversity, (g—i) Pielou’s evenness]. Adjusted *P* values based on Tukey pair-wise testing of estimated marginal mean contrasts.

Taxon richness, Shannon diversity, and Pielou’s evenness indices were calculated at the family level. Model results indicated a significant interaction between bog and sampling date for all three alpha diversity metrics. In all three bogs, all three alpha diversity metrics were significantly higher in September than earlier sampling dates (Fig 2). Richness increased significantly between May and September for Beckman bog samples (mean = 49.1 versus 78.3, SE = 7.41 and 7.41, t = -2.787, *P* < 0.05) and for BBSR samples (mean = 51.1 versus 80.8, SE = 6.80 and 7.09, t = -3.143, *P* < 0.05). For Clearwater samples, richness decreased between May and July (mean = 84.5 versus 48.9, SE = 5.77 and 5.79, t = 4.384, *P* < 0.001), as well between May and August (mean = 84.5 versus 59.0, SE = 5.77 and 5.75, t = 3.137, *P* < 0.05). After its low mark in July, Clearwater sample richness rose significantly from July to September (mean = 48.9 versus 88.7, SE = 5.79 and 5.75, t = -4.846; *P* < 0.001), as well as between August and September (mean = 59.0 versus 88.7, SE = 5.75 and 5.75, t = -3.643, *P* < 0.05).

A similar pattern was observed for Shannon diversity, with significant increases between May and September (mean = 2.35 versus 3.15, SE = 0.169 and 0.169, t = -3.319, *P* < 0.01) in Beckman samples, as well as in BBSR samples (mean = 2.29 versus 3.30, SE = 0.155 and 0.162, t = -4.074, *P* < 0.001). Shannon diversity also increased significantly from May to July (mean = 2.29 versus 2.88, SE = 0.155 and 0.166) (t = -2.733; *P* < 0.05) and August to September (mean = 2.51 versus 3.30, SE = 0.156 and 0.162, t = -3.671; *P* < 0.01) across BBSR populations. Clearwater sample Shannon diversity decreased between May and August (mean = 3.07 versus 2.54, SE = 0.132 and 0.131, t = 2.849; *P* < 0.05), but rose significantly between July and September (mean = 2.63 versus 3.20, SE = 0.132 and 0.131, t = -3.045; *P* < 0.05), as well as from August to September (mean = 2.54 versus 3.20, SE = 0.131 and 0.131, t = -3.554, *P* < 0.01).

Finally, Pielou’s evenness index rose between May and September (mean = 0.613 and 0.723, SE = 0.029 and 0.029, t = -2.748, *P* < 0.05) in Beckman and in BBSR (mean = 0.582 versus 0.757, SE = 0.026 and 0.027, t = -4.658, *P* < 0.001). Furthermore, significant increases were also observed between May and July (mean = 0.582 and 0.715, SE = 0.026 and 0.028, t = -3.648, *P* < 0.01) and August and September (mean = 0.621 versus 0.751, SE = 0.027 and 0.027, t = -3.569, *P* < 0.05) in BBSR samples. Evenness in Clearwater bog samples fluctuated the least, with only a significant increase detected between August and September (mean = 0.624 versus 0.715, SE = 0.022 and 0.022, t = -2.890, *P* < 0.05).

### Beta diversity

**Fig 3:**
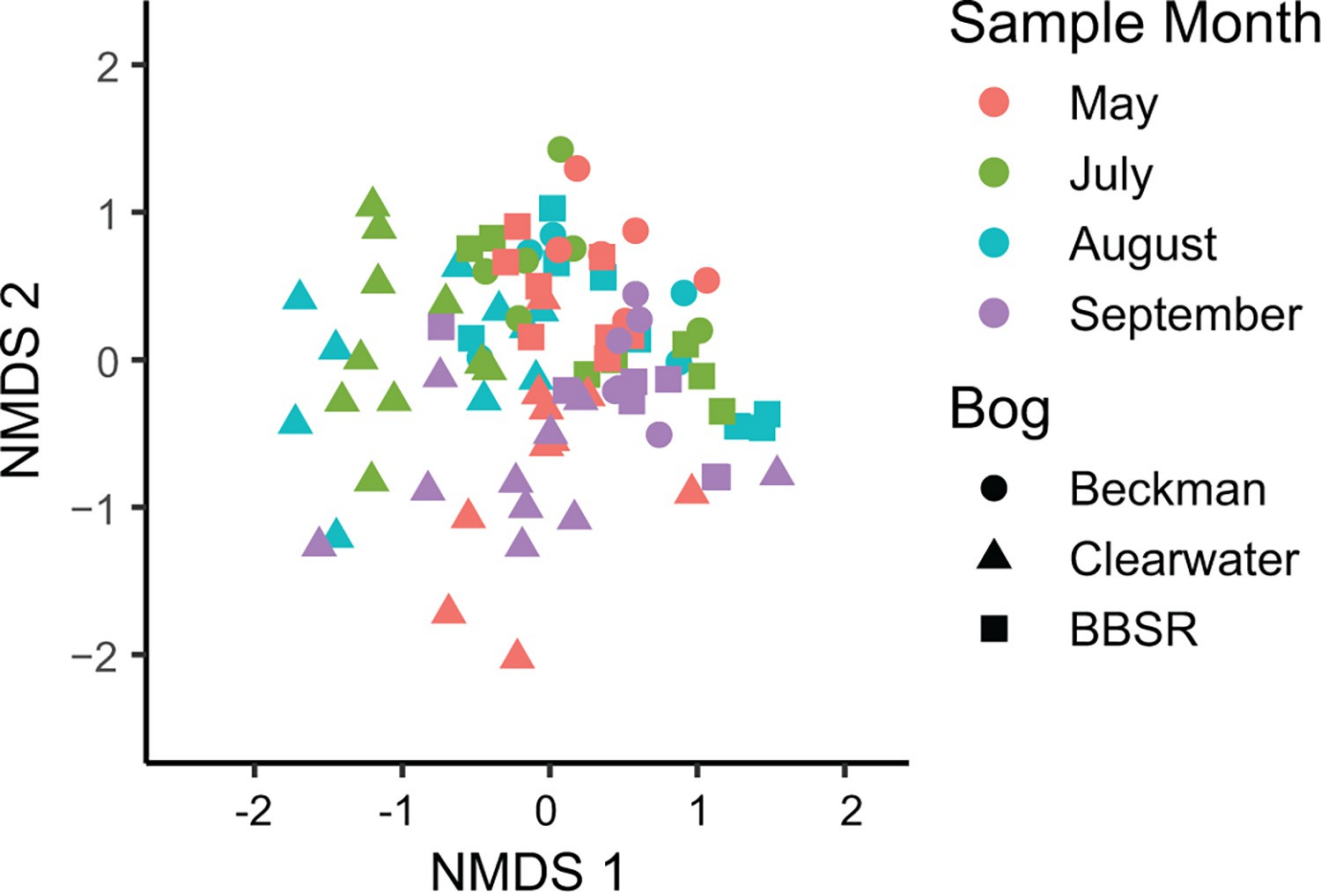
NMDS Ordination at the ASV level (stress = 0. 0.087; k = 5).

Ordination analysis demonstrated significant clustering of samples by sample date (PERMANOVA R^2^ = 0.081; *P* = 0.001) and bog location (R^2^ = 0.090; *P* = 0.001), as well as their interaction (R^2^ = 0.092; *P* = 0.001) (Table 2 and Fig 3). However, significant differences in beta-dispersion between bogs and sample dates were noted, which could also explain the significant differences in PERMANOVA results. Individual pitcher identity accounted for the greatest partition of family composition variability (R^2^ = 0.146), but this value was not statistically significant (*P* = 0.800).

**Table 2:** Phytotelm bacteriome PERMANOVA results (beta diversity).

***Formula***: Distance ~ Sample Date * Bog + Pitcher Age + Plant identity +					
*Pitcher identity + Amplicon copies*				* *	* *
	**DF**	**R** ^**2**^	**F**	**p (>F)**	
Sample Date	3	0.081	3.127	0.001	
Bog	2	0.090	5.262	0.001	
Pitcher age	1	0.012	1.448	0.039	
Plant identity	3	0.057	2.227	0.001	
Pitcher identity	18	0.146	0.946	0.800	
Amplicon copies	1	0.015	1.763	0.007	
Sample Date:Bog	6	0.092	1.791	0.001	
Residual	59	0.506			
Total	93	1.000			

### Relative family abundance and prevalence

**Fig 4:**
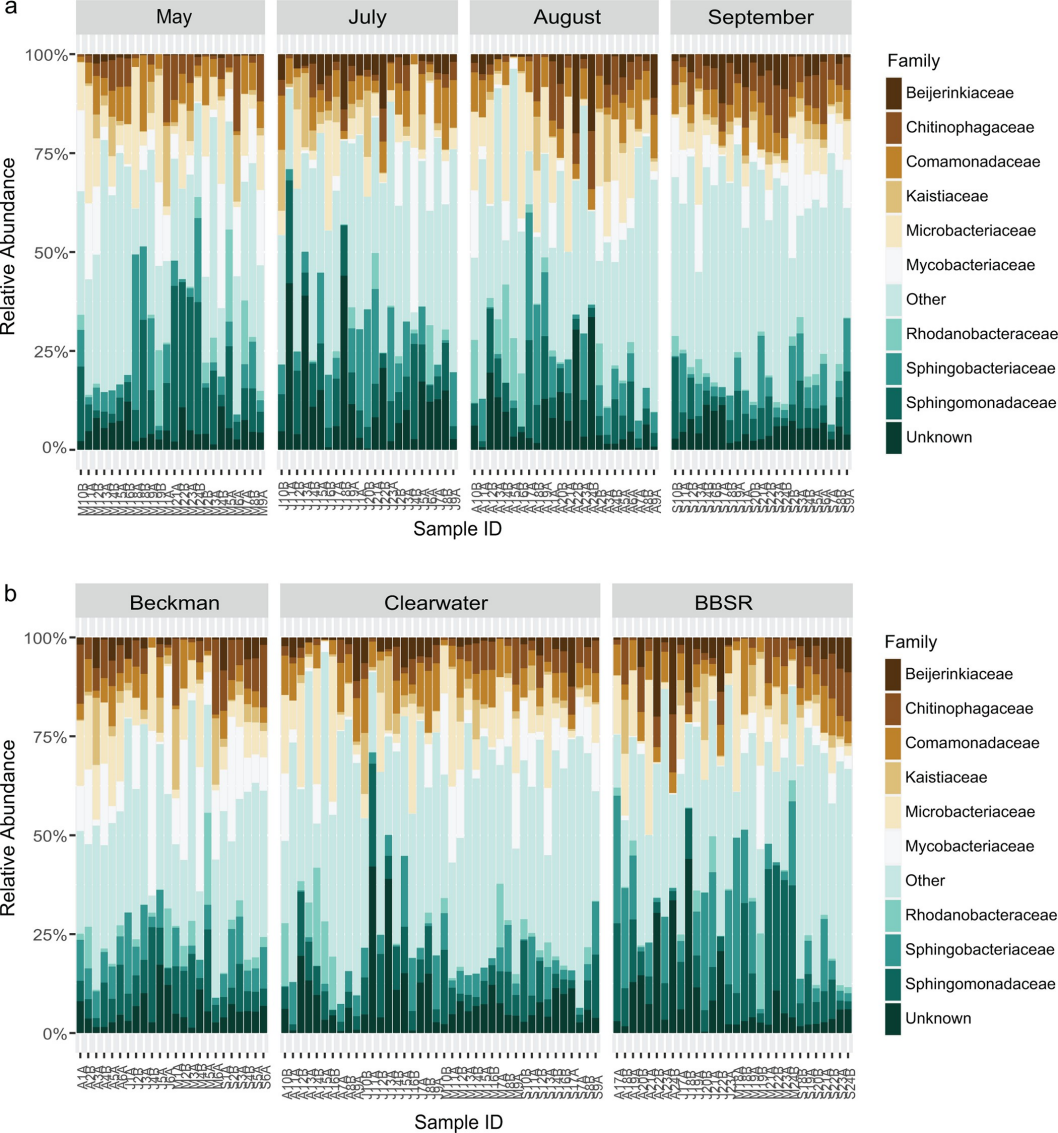
Family-level relative abundance. Each bar represents one sample, and samples are grouped by (a) sample date and (b) bog location.

The 11 most abundant families across all samples in this study accounted for >75% of all family-level read counts, with Microbacteriaceae, Sphingomonadaceae, Sphingobacteriaceae, Mycobacteriaceae and Comamonadaceae representing nearly 40% of all family-level read counts (Fig 4 and S1 Table). Microbacteriaceae, Sphingomonadaceae, and Comamonadaceae were present in every sample, and an additional twelve families were present in at least 90% of samples.

**Fig 5:**
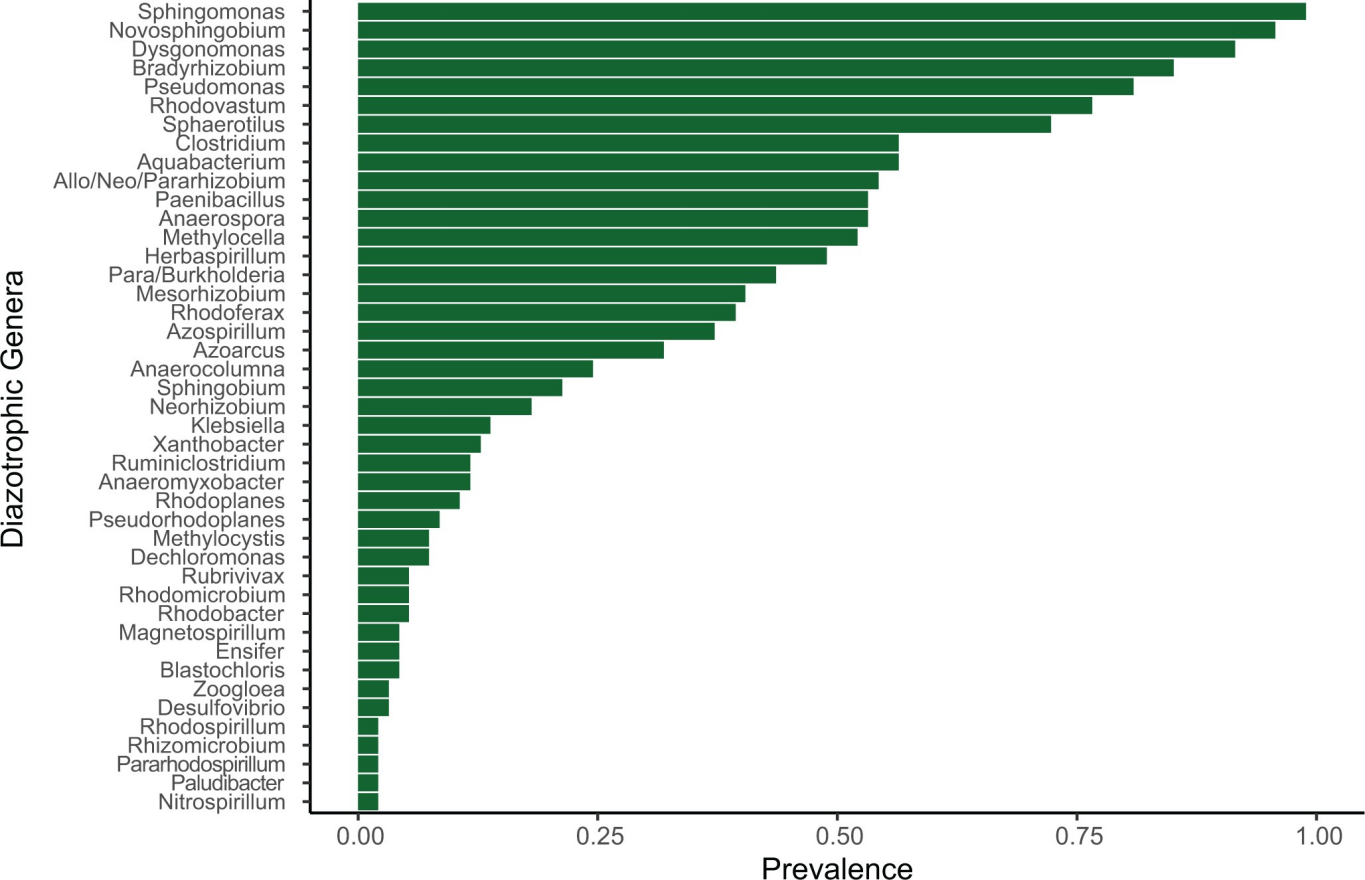
Prevalence of diazotroph-containing genera in *S*. *purpurea* phytotelm samples. Genera present in at least three samples (prevalence > 0.02) are shown.

Forty-eight genera documented to contain diazotrophic species were also identified in the phytotelm samples of this study (Fig 5 and S2 Table). These genera represent a wide phylogenetic range from 30 families, with *Sphingomonas*, *Novosphingobium*, *Dysgonomonas* and *Bradyrhizobium* each present in over 85% of samples. An additional 10 diazotroph-containing genera were present in at least 50% of the samples.

### Differential abundance testing

**Fig 6:**
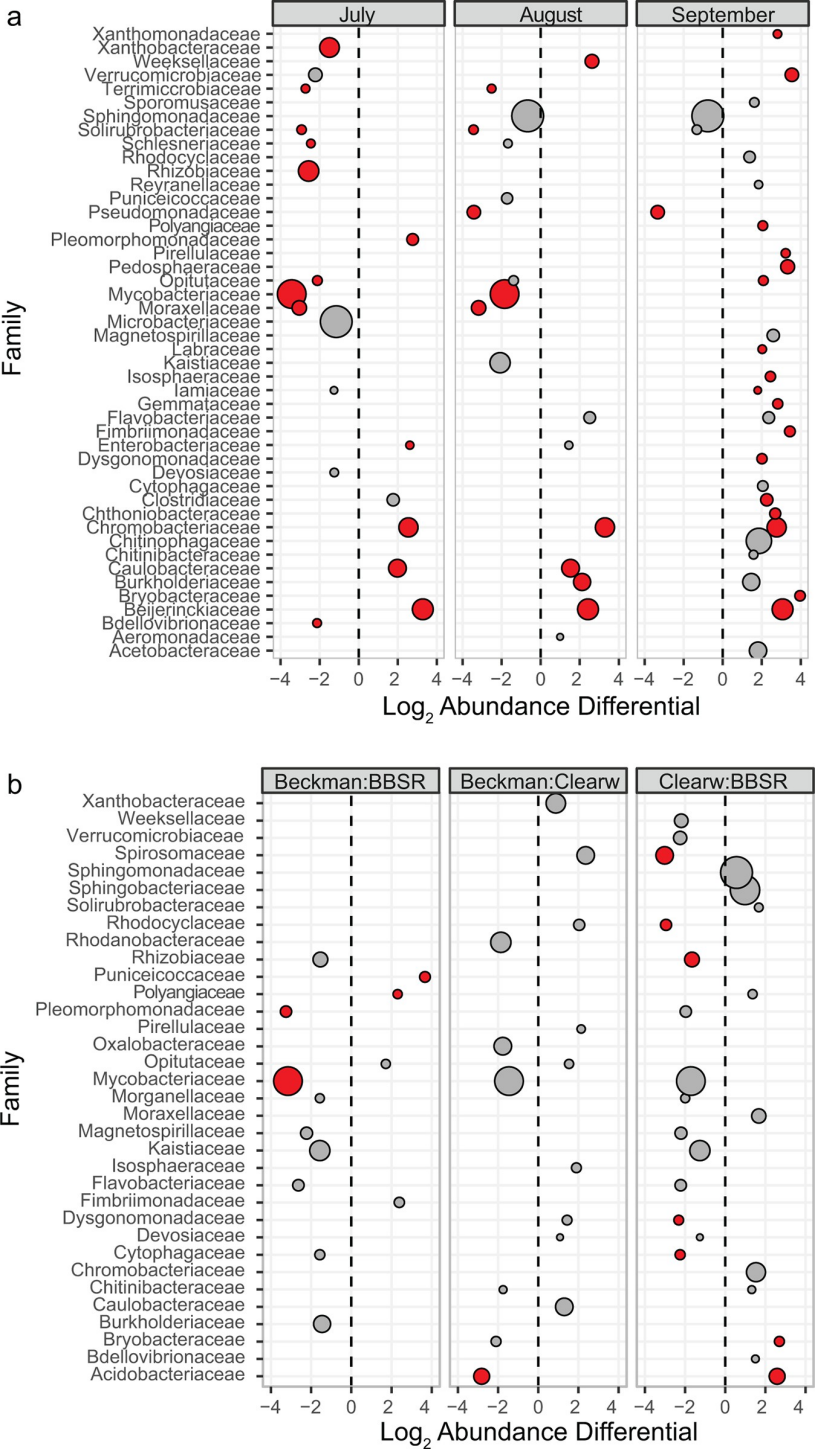
(a) Pair-wise differential relative abundance by sample date (comparisons to May), and (b) by bog location. Red bubbles indicate differential abundance q-value < 0.05; bubble size indicates relative mean abundance. Only families with mean relative abundance > 0.1% shown.

Of the families with the greatest relative abundances across all samples, Beijerinckiaceae, Caulobacteriaceae, and Chromobacteriaceae had significant increases in relative abundance (absolute value of Log_2_ change > 1.0; q-value < 0.05) in July when compared to May, while Xanthobacteraceae, Rhizobiaceae and Mycobacteriaceae diminished significantly during that period (Fig 6). Overall, 17 families exhibited significant abundance increases in September when compared to May, with only the pseudomonads significantly decreasing in relative abundance by September.

There were few significant changes in family-level relative abundances in Beckman bog samples when compared to BBSR. Declines in abundance were significant for both Pleomorphomonadaceae and Mycobacteriaceae, as were increases in Puniceicoccaceae and Polyangiaceae. Only the Acidobacteriaceae showed a significant abundance decline in Clearwater samples when compared to those of Beckman. However, the bacterial communities of the northernmost BBSR samples had significantly reduced relative abundances of Spirosomaceae, Rhodocylaceae, Rhizobiaceae, Dysgonomonadaceae and Cytophagaceae when compared to Clearwater sample, while Acidobacteriaceae and Bryobacteraceae had increased relative abundance.

### nifH gene quantification

**Fig 7:**
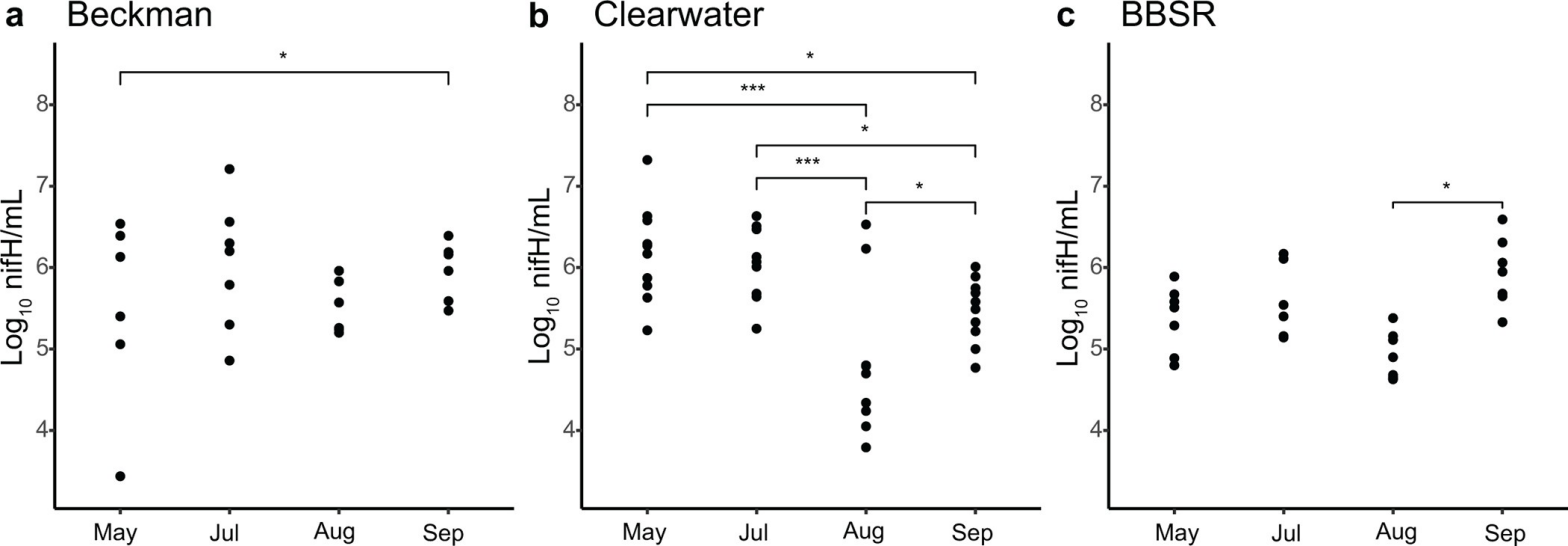
Log_10_ nifH gene copies per mL *S*. *purpurea* phytotelm fluid by sample month for bogs (a) Beckman, (b) Clearwater, and (c) BBSR. *P* values (adjusted) based on Tukey pair-wise testing of estimated marginal mean contrasts.

Bacterial nifH genes were detected by qPCR in 100% (n = 94) of *S*. *purpurea* phytotelm samples, with a median of 5.63 log_10_ nifH copies/mL phytotelm fluid, and a range of 3.44 to 7.32 log_10_ nifH copies/mL phytotelm fluid (Fig 7). Mixed effects model results indicated a significant interaction between bog and sampling date for nifH concentration. In Beckman bog populations, nifH concentrations remained stable throughout the sampling period, with no significant differences in pairwise estimated marginal mean contrasts. However, nifH concentrations in Clearwater samples were stable from May to July (mean = 6.18 and 6.15, SE = 0.178 and 0.179, t = 0.140, *P* > 0.99) before significantly decreasing between July and August (mean = 6.15 versus 4.81, SE = 0.179 and 0.178, t = 5.690, *P* < 0.001), and rebounding from August to September (mean = 4.81 versus 5.51, SE = 0.178 and 0.178, t = -2.955, *P* < 0.05). In addition, Clearwater sample contrasts of mean nifH concentration were also significant between May and August (mean = 6.18 versus 4.81, SE = 0.178 and 0.178, t = 5.840, *P* < 0.001), May and September (mean = 6.18 versus 5.51, SE = 0.178 and 0.178, t = 2.845, *P* < 0.05), and July and September (mean = 6.15 versus 5.51, SE = 0.179 and 0.178, t = 2.694, *P* < 0.05). In BBSR samples, a similar pattern of declining nifH concentration was observed between July and August (mean = 5.69 versus 5.12, SE = 0.228 and 0.215, t = -1.160, *P* > 0.5), but was not statistically significant. As in the Clearwater samples, however, nifH concentrations increased significantly between August and September (mean = 5.12 versus 6.02, SE = 0.215 and 0.220, t = -3.230, *P* < 0.05). Pairwise comparison of nifH concentrations between bogs indicated only significant difference between the two southernmost bogs, Beckman and Clearwater, respectively, in May (mean = 5.47 versus 6.18, SE = 0.229 and 0.178, t = -2.457, *P* < 0.05) and again in August (mean = 5.55 versus 4.81, SE = 0.230 and 0.178, t = 2.575, *P* <0 .05).

Mixed effects modeling also indicated a significant association between nifH qPCR copy concentration and 16S rDNA qPCR copy number (Est. = 0.164, SE = 0.076, t = 2.165, *P* < 0.05) and pitcher age (Est. -0.328, SE = 0.157, t = -2.089, *P* < 0.05), respectively.

## Discussion

The complex structure and ecology of phytotelm microbial communities in insect-trapping pitcher plants of family Sarracenaceae have been the subject of study for decades. Metagenome analyses have identified numerous environmental factors involved in the recruitment and succession of these communities. Host plant species within the genus *Sarracenia*, ambient temperature, captured prey species, and the array of inquiline eukaryotic predators housed by these plants have all been shown to influence the phytotelm microbiome [10,16,19,53–56]. Furthermore, the geographic location of host plants at the continental level also affects these communities through various correlated factors, such as climate and substrate composition [57,58].

Within the region of this study, we observed that bog location of *S*. *purpurea* plants had only minimal impact on the alpha diversity of phytotelm prokaryote communities. This was not surprising since the peatland/bog systems of this part of *S*. *purpurea*’s range exhibit similar conditions, but differ substantially from the plant’s habitat in the eastern and southeastern United States. These differences include not only climate, but the associated biotic community and invertebrate prey species available to *Sarracenia* pitchers. In contrast, these bacterial communities did exhibit changes in alpha diversity metrics over the course of the sampling period, which approximates the growing season in this region. With the exception of the May sampling date in Clearwater bog samples, significant increases in family richness, Shannon diversity, and Pielou’s evenness indices were observed between the early dates and September, with the most dramatic variations occurring from July and August to September. This pattern was similar in each bog location. Average daily temperatures during the sampling period increased at all sites from May until late July (S4 Table). Temperature likely affects microbial growth in these communities, as well as prey activity [8,18,56]. Interestingly, the most statistically significant changes in phytotelm alpha diversity occurred as average daily temperatures diminish late in the growing season.

Beta diversity was significantly different by both bog location and sample date, although the amount of overall variance partitioned to these two variables was relatively low at a combined 17% (Table 2). Conversely, individual pitcher identity explained a large (R^2^ = 0.146) but statistically insignificant (*P* = 0.800) amount of variability in beta diversity. The large R^2^- and *P*-values associated with pitcher identity could result from a combination of high biological importance, but a relatively small number of observations per pitcher. Future researchers of these phytotelm ecosystems can offset at least some of this “pitcher identity” effect by increasing the number of observations per leaf, as well as the number of leaves and plants sampled, and by using metabarcoding of eukaryotic DNA to profile the prey species contained within each individual leaf. The prokaryotes identified within an individual pitcher may be driven primarily by the microorganisms inhabiting arthropod prey that happen to fall into that phytotelma, causing different pitchers on a single plant to harbor significantly different bacteriomes [18,59]. Under this hypothesis, “pitcher identity” would be an important contributor to microbiome composition because it would represent the unique eukaryotic and prokaryotic organisms that happened to be captured by each individual leaf. Future researchers of these phytotelm ecosystems can offset at least some of this “pitcher identity” effect by increasing the number of leaves and plants samples, and by using metabarcoding of eukaryotic DNA to profile the prey species contained within each individual leaf.

We speculated that agricultural land use practices near the Clearwater and Beckman bogs might influence *S*. *purpurea* phytotelm bacteriome diversity in comparison to those of the more isolated BBSR bog. Anthropogenic stressors such as tillage, soil fertilization, and herbicide/pesticide runoff can produce a litany of ecological changes to aquatic ecosystem. While these and other potential stressors may account for some variation between different bog locations, no obvious pattern was observed between BBSR and the bogs with nearby agricultural lands. If such land use practices do impact phytotelm microbiomes, myriad biological mechanisms may buffer the *S*. *purpurea* phytotelm bacteriome from dramatic shifts in diversity. In fact, the phytotelm bacteriomes in all three bog locations exhibited similar patterns of both alpha and beta diversity metrics. This finding is consistent with a recent report that showed that alpha diversity changes progressively over a much longer, multi-continent, latitude transect, but does so gradually [58].

### Comparison with other 16S metagenome studies

Although several researchers have characterized the bacteriome *of S*. *purpurea* phytotelmata in recent decades, these culture-based analyses are difficult to compare with those employing modern metagenome sequencing methods. Many of the phyla found in previous, culture-based studies [9,13–16] were also found in the samples of this study. A more appropriate comparison can be made between our results and those described in a recent metagenome analysis of the *S*. *purpurea* phytotelm microbiome in two bogs in Wisconsin, USA, by Grothjan, et. al., [17]. Grothjan’s group analyzed fewer phytotelm samples (n = 10) and identified 49 families with relative abundances greater than 0.01%, while our analysis found 118 families meeting this criterion in 94 samples. Many families with the greatest relative abundances were common to both studies. These included Burkholderiaceae, Caulobacteraciae, Comamonadaceae, Microbacteriaceae, Moraxellaceae, Mycobacteriaceae, Oxalobacteriaceae, Rhizobiaceae, Sphingobacteriaceae, and Sphingomonadaceae, all of which had abundances greater than 1.0% in both studies. Coxiellaceae and Rhodospirillaceae had negligible abundances in the Minnesota samples, but represented the greatest proportion of the Wisconsin study (15.7% and 10.0%, respectively). Grothjan also reported significant and distinctive beta-diversity differences between the two sample site communities, which was markedly different from our findings in the three Minnesota sites.

### Resident diazotrophic microbes may contribute to the *S*. *purpurea* nitrogen budget

We observed the widespread presence of taxonomically diverse diazotrophic genera in *S*. *purpurea* phytotelmata in the study region and throughout the sampling period. Furthermore, bacterial nifH genes were detected at biologically significant concentrations in all phytotelm samples of this study, with a median value of 5.63 log_10_ nifH copies/mL phytotelm fluid. In addition, the concentration of nifH genes in our samples was remarkably stable across sample (bog) locations. The only statistically significant differences between estimated marginal means of nifH concentration were found between sample months. However, with the exception of a slight drop in August, nifH concentrations averaged above 5.00 log_10_ copies/mL phytotelm fluid during each sample month. Therefore, these relatively minor fluctuations may not be biologically significant. These results are also consistent with previous findings of inquiline diazotrophs in the phytotelm fluid of pitchers from the Nepthenthaceae, as well as the rhizome tissue of *Sarracenia* species [34,35]. While this study did not analyze nitrogen fixation activity within phytotelm fluid per se, our findings collectively support the hypothesis that diazotrophs are a key component of *S*. *purpurea* phytotelm communities and may contribute to the host plant’s bioavailable nitrogen requirements. Further research exploring the degree of nifH gene expression in *S*. *purpurea* phytotelm fluid, as well as chemical analysis of nitrogen fixation is being planned.

The insectivory of *Sarracenia purpurea* and its congeners is well-established as a supply mechanism for scarce nutrients, including biologically available nitrogen compounds [3,7,26,60,61]. Researchers have shown much of the digestive enzyme milieu that degrades trapped phytotelm arthropods is generated by the bacterial community. This has strongly suggested that *S*. *purpurea* and its microbial inhabitants exist in a mutualistic association. Until recently, less attention has been given to the potential role of resident nitrogen-fixing prokaryotes in this relationship.

The presence of diazotrophs and their nitrogenase systems in all *S*. *purpurea* samples in this study may be ecologically coincidental. However, it is more likely that nitrogen fixation by resident prokaryotes represents another mutually beneficial tool in the complex nutrient acquisition repertoire of *S*. *purpurea*. Moreover, the diversity of diazotroph-bearing genera in these samples, as well as their consistent abundances and prevalences throughout both the study sites and growing season, suggest that the collective metabolic function of nitrogen fixation may be more critical than which diazotrophic taxa are present [62,63]. Further studies are needed to determine whether a core diazotrophic community in *Sarracenia* phytotelmata exists, or if nitrogen fixation is a consistent metabolic necessity for *Sarracenia* that is produced a shifting community of phytotelm diazotrophs.

The paradigm of *Sarracenia* species as predators has gradually evolved to a more sophisticated understanding that these plants are indeed insectivores, but also exemplars of microbial aquaculture. Our findings offer further evidence that nitrogen acquisition for pitcher plants is a multi-layered, complex system in which diazotrophs play a contributing role, and that their presence and diversity remain regionally and temporally constant in *S*. *purpurea* phytotelmata, in at least part of its geographic range. We are currently exploring the phylogeny and diversity of the nifH genes found in the phytotelm fluid of *S*. *purpurea* in this and other regions of the plant’s range. However, more research is needed to elucidate the relative contributions of bioavailable nitrogen not only from degrading captured prey, but also from the metabolic processes of the inquiline bacterial community.

## Supporting information

S1 Table Average family relative abundance and prevalence for all Sarracenia purpurea phytotelma samples. (A) Relative family abundance; (B) Family prevalence.
(XLSX)

S2 Table Average prevalence of genera with documented diazotrophic species; all Sarracenia purpurea phytotelmata samples. (XLSX)

S3 Table Bacterial nifH gene concentrations (Log10 nifH genes/mL phytotelm fluid). (XLSX)

S4 Table Average daily temperatures (F) at nearest National Weather Service Stations to sample locations. (XLSX)
